# The factors of deterioration in long-term clinical course of lumbar spinal canal stenosis after successful conservative treatment

**DOI:** 10.1186/s13018-018-0947-2

**Published:** 2018-09-18

**Authors:** Masanori Tsubosaka, Shuichi Kaneyama, Tomonori Yano, Koichi Kasahara, Aritetsu Kanemura, Masato Takabatake, Hiroaki Hirata, Masatoshi Sumi

**Affiliations:** 1 Department of Orthopaedic Surgery, Kobe Rosai Hospital, 4-1-23, Kagoike-dori, Chuo-ku, Kobe, 651-0053 Japan; 2 Department of Orthopaedic Surgery, Hyogo Rehabilitation Centre Central Hospital, 1070 Akebono-cho, Nishi-ku, Kobe, 651-2181 Japan

**Keywords:** Lumbar spinal canal stenosis, Conservative treatment, Prognosis, Prognostic factors

## Abstract

**Background:**

The treatment of lumbar spinal canal stenosis (LSS) depends on symptom severity. In the absence of severe symptoms such as severe motor disturbances or bowel and/or urinary dysfunction, conservative treatment is generally the first choice for the treatment of LSS. However, we experienced cases of worsening symptoms even after successful conservative treatment. The purpose of this study is to investigate the long-term clinical course of LSS following successful conservative treatment and analyze the prognostic factors associated with symptom deterioration.

**Methods:**

The study included 60 LSS patients (34 females and 26 males) whose symptoms were relieved by conservative treatment between April 2007 and March 2010 and who were followed up for 5 years or longer. The mean age at admission was 64.8 ± 8.5 years (range, 40–85 years old), and the mean follow-up period was 7.3 years (range, 5.8–9.5 years). We defined “deterioration” of symptoms as the shortening of intermittent claudication more than 50 m compared with those at discharge or the occurrence or progression of lower limb paralysis, and “poor outcome” as the deterioration within 5 years after discharge. The long-term outcome of conservative treatment for LSS was analyzed by Kaplan-Meier analysis. Furthermore, logistic regression analysis was performed to reveal the risk factors of poor outcome for clinical classification, severe intermittent claudication (≤ 100 m), lower limb muscle weakness, vertebral body slip (≥ 3 mm), scoliosis (Cobb angle ≥ 10°), block on myelography, and redundant nerve roots of the cauda equina.

**Results:**

Thirty-four (56.7%) patients preserved their condition at discharge during the follow-up, whereas 26 patients (43.3%) showed deterioration. Sixteen patients had a decreased intermittent claudication distance, and 10 patients had newly developed or progressive paralysis. The probability of preservation was maintained at 68.3% at 5 years after discharge. Logistic regression analysis demonstrated that only severe intermittent claudication (≤ 100 m) was a significant risk factor of a poor outcome (*p* = 0.005, odds ratio = 6.665).

**Conclusions:**

The patients with severe intermittent claudication should be carefully followed up because those are the significant deterioration candidates despite the success in conservative treatment.

## Background

Lumbar spinal canal stenosis (LSS) is a disease characterized by one or several symptoms such as low back pain, pain and/or numbness in the lower extremities, and neurogenic claudication that occurs secondary to spinal canal narrowing [1]. The treatment of LSS depends on symptom severity. The major indications for surgery are as follows: intolerable pain in daily activities despite adequate conservative treatment, progressively limited walking distance or standing endurance, and major neural deficits or evolving/progressive neural damage such as cauda equina syndrome, drop foot, and bowel and/or urinary dysfunction [2–6]. On the other hand, in the absence of severe symptoms such as severe motor disturbances or bowel and/or urinary dysfunction, conservative treatment is generally the first choice for the treatment of LSS [7–10]. Components of conservative treatment vary but generally include bed rest, medication or injection of anti-inflammatory analgesics, physical therapy or reduction of postural lordosis, and muscle weakness treatment with reportedly good outcomes [9, 11–13]. However, we experienced cases of worsening symptoms even after successful conservative treatment. Although there have been several reports regarding prognostic factors for the conservative treatment of LSS, studies with long-term follow-up have rarely been reported [9, 14, 15]. In addition, these reports did not describe the time course of the progress of symptom deterioration: when and how the symptom deteriorated in the patients with negative factors after successful conservative treatment in spite of its importance for decision-making around treatment. This study aimed to investigate the clinical course and prognosis of LSS after successful conservative treatment and identify the prognostic factors for predicting aggravation risk.

## Methods

### Study population

All patients were examined by at least two physicians and diagnosed with LSS based on the symptoms (back, buttock, or leg pain and/or numbness; intermittent claudication or muscle weakness on legs), physical findings, and radiological findings demonstrating lumbar spinal canal stenosis of corresponding levels. We performed inpatient intensive conservative treatment described in the other sections if the patients had difficulty in normal daily activity in their home suffering from the symptoms which had prolonged despite outpatient conservative treatment including administration of nonsteroidal anti-inflammatory drugs, application of elastic lumbar orthosis and/or caudal block.

This study was designed to include the patients who had achieved symptom relief with the inpatient intensive conservative treatment between April 2007 and March 2010 and were followed up for 5 years or longer, and excluded those who were diagnosed with lumbar disc herniation, had osteoarthritis of the knee or hip joint, had cerebrovascular diseases, or had undergone previous spinal surgery.

Among 73 patients successively treated by inpatient intensive conservative treatment, 60 patients (34 females and 26 males) satisfied the criteria. The mean age at admission was 64.8 ± 8.5 years (range, 40–85 years old), and the mean follow-up period was 7.3 years (range, 5.8–9.5 years). The patients were clinically classified into three types as described by Miyamoto et al. [8] according to subjective symptoms and objective findings. The myelographic findings were taken into account in the objective findings. The myelographic findings were classified into three groups: completely blocked centrally (6 cases), centrally blocked but not completely (19 cases), and root defect (35 cases). Furthermore, the presence or absence of redundant nerve roots of the cauda equina was also considered (presence, 11 cases; absence, 49 cases). In consideration of these objective findings in addition to the subjective findings, the classification was carried out in three types as follows: radicular, 39 cases; cauda equina, 12 cases; and mixed, 9 cases. The hospital ethics committee approved the study protocol, and the patients provided informed consent for participation.

### Inpatient intensive conservative treatment

Our protocol for inpatient conservative treatment consisted of pelvic traction, the application of a body cast, and an epidural steroid injection such as an epidural or selective nerve root block. Conservative treatment was performed in a phased manner from least invasive (pelvic traction) to most invasive (selective nerve root block) until the symptoms improved. Patients were normally admitted for 2–3 weeks. Pelvic traction was used 8 h a day to force patients into a jack-knife position and was applied for 2–3 days as a guide. The traction force was also applied one third of his/her weight. If pelvic traction was ineffective, a body cast was applied for 2 or 3 days to force the patient’s lumbar spine into a slightly flexed position. The reason for adopting a slightly flexed position of the lumbar spine was that it was reported that the cross-sectional area of the spinal canal had been shown to decrease with extension and increase with flexion [16]. If the body cast was effective, a Williams corset was occasionally fitted after discharge. The patients were asked to wear a Williams corset mainly when going out during lifetime. An epidural block (10.0 mL of 1.0% xylocaine mixed with 10.0 mg of water-soluble predonine) was injected into the epidural space a maximum of two times. A selective nerve root block (1.5 mL of 1.0% xylocaine mixed with 10.0 mg of water-soluble predonine) was administered around the epiradicular membrane under fluoroscopy guidance for radicular pain a maximum of two times.

### Outcome evaluation

The patients were routinely followed up with the physical or radiographic examination in the outpatient clinic after discharge. The physical or radiographic examination follow-up timings were 3, 6, and 12 months and every year after discharge. During follow-up, the symptoms were considered to deteriorate if the patient presented a shortening intermittent claudication distance more than 50 m and/or had a worse neurological defect than that at discharge (deterioration group). The neurological defect was defined as a drop of manual muscle test more than one grade. Symptoms were considered preserved if the patient showed no deterioration (preservation group). An outcome was defined as poor when the patient experienced deterioration by 5 years post-discharge. The long-term outcomes of conservative treatment for LSS were assessed by Kaplan-Meier analysis. The patients were stratified according to the following factors: age (≤ 65 years or > 65 years), severe intermittent claudication at the initial visit (≤ 100 m), clinical classification (radicular type or cauda equina and mixed type), lower limb muscle weakness, vertebral body slip (≥ 3 mm), scoliosis (Cobb angle ≥ 10°), block on myelography, and redundant nerve roots of the cauda equina. The vertebral body slip was assessed on lateral radiographs of the lumbar spine and measured the sagittal distance of the slipped vertebral body. The redundant nerve root means the redundancy of cauda equina. Differences in outcomes were analyzed in each group by the log-rank test. Furthermore, the risk factors incurring a poor outcome even after successful conservative treatment for LSS were analyzed using logistic regression analysis of the factors listed above. Differences of age, sex, clinical LSS classification, the presence or absence of intermittent claudication, muscle weakness, vertebral body slip, scoliosis, block on myelography, and redundant nerve root between the preservation group and the deterioration group were also analyzed by the chi-square test. *p* values < 0.05 were considered statistically significant.

## Results

**Fig. 1:**
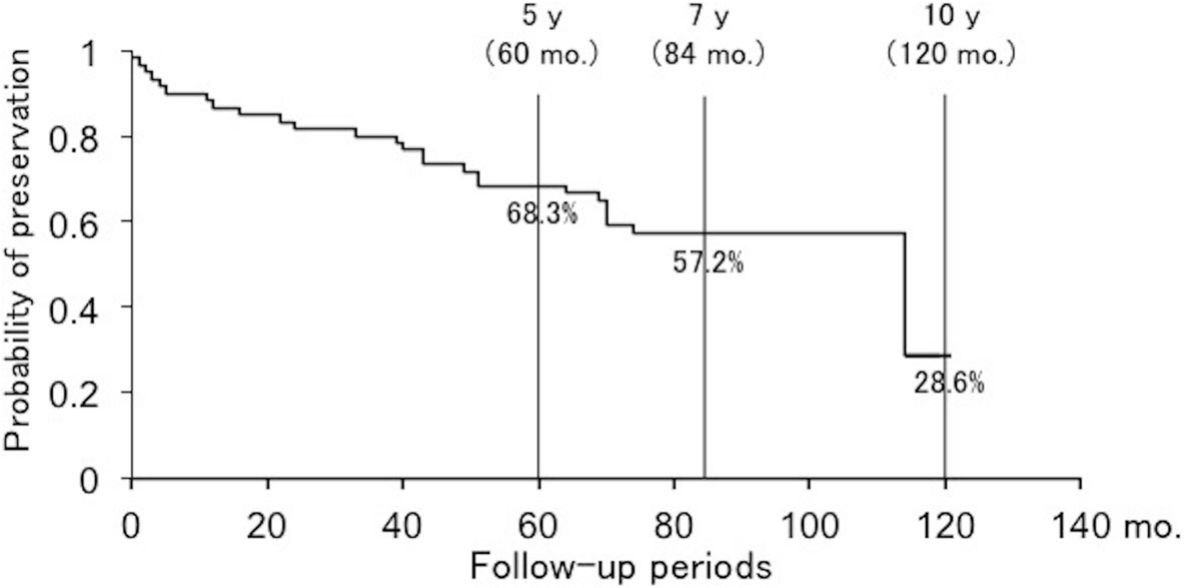
Long-term results of conservative treatment for LSS in this study. The probability of symptom preservation after the successful conservative treatment of LSS remained at 68.3% 5 years post-discharge and 57.2% 7 years post-discharge

**Fig. 2:**
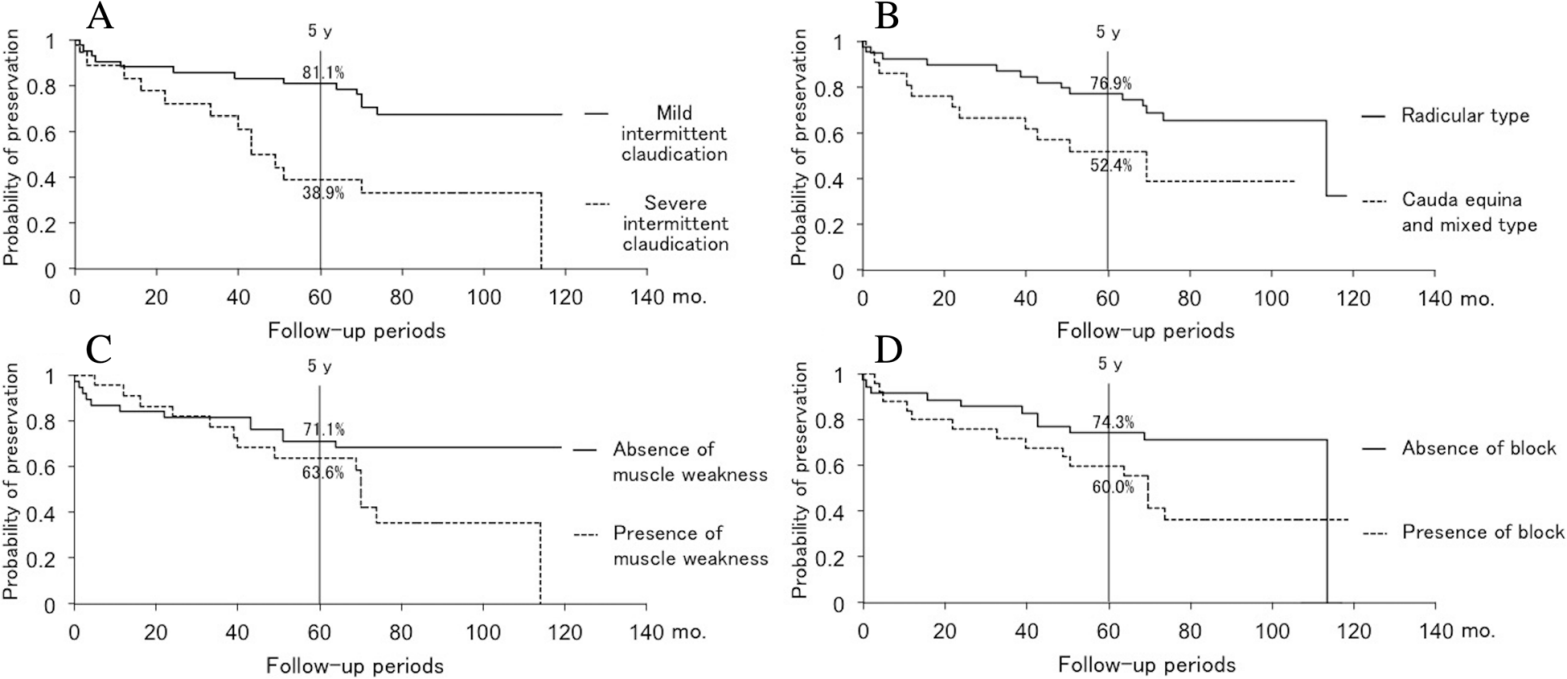
**a** Comparison of symptom preservation in patients with severe versus mild intermittent claudication using Kaplan-Meier analysis. The outcomes of conservative treatment were worse in patients with severe intermittent claudication at the initial visit (≤ 100 m) than those in patients with mild intermittent claudication (between 100 and 500 m) (*p* = 0.004). **b** Comparison of symptom preservation in patients with radicular versus cauda equina or mixed type using Kaplan-Meier analysis. The outcomes of conservative treatment were worse in patients with cauda equina or mixed-type LSS than those in patients with radicular-type LSS (*p* = 0.039). **c** Comparison of symptom preservation in patients with versus without lower limb weakness using Kaplan-Meier analysis. The outcomes of conservative treatment were worse in patients with lower limb muscle weakness than those in patients with no muscle weakness (*p* = 0.049). **d** Comparison of symptom preservation by absence or presence of block on myelography using Kaplan-Meier analysis. The outcomes of conservative treatment were worse in patients with block on myelography than those in patients with no block on myelography (*p* = 0.045)

**Fig. 3:**
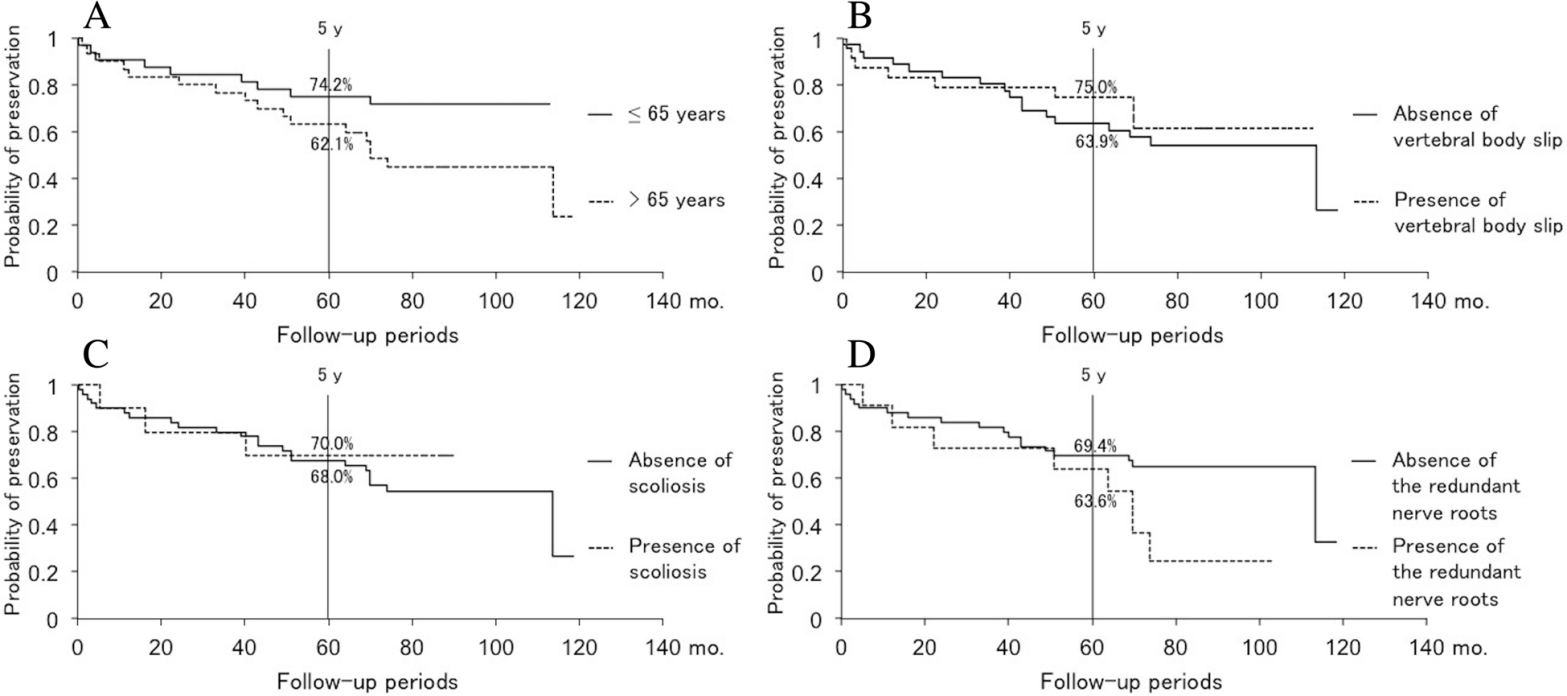
**a** Comparison of symptom preservation in patients ≤ 65 versus > 65 years of age using Kaplan-Meier analysis. Age did not significantly influence symptom deterioration. **b** Comparison of symptom preservation in patients with versus without vertebral body slip using Kaplan-Meier analysis. Vertebral body slip (≥ 3 mm) did not significantly influence symptom deterioration. **c** Comparison of symptom preservation in patients with versus without scoliosis using Kaplan-Meier analysis. Scoliosis (Cobb angle ≥ 10°) did not significantly influence symptom deterioration. **d** Comparison of symptom preservation in patients with the presence or absence of redundant nerve roots of the cauda equina Kaplan-Meier analysis. Redundant nerve roots of the cauda equina did not significantly influence symptom deterioration

Thirty-four patients (56.7%) preserved the conditions at discharge until the latest follow-up, while 26 patients (43.3%) showed deterioration during the follow-up period. The average age of the patients who showed deterioration during the follow-up period (67.8 ± 6.9 years) was significantly higher than that of the patients whose conditions at discharge were preserved (62.5 ± 8.8 years). Furthermore, the patients with severe intermittent claudication, muscle weakness, and redundant nerve root tend to be significantly worse of their symptoms. On the other hand, there were no significant differences in sex, clinical LSS classification and the presence of vertebral body slip, scoliosis, and block of myelography between the preservation group and deterioration group (Table 1). In the preservation group, 3 patients had no symptoms, 20 patients had minor symptoms such as slight low back pain or numbness of the lower extremities, and 11 patients needed only medication therapy at the latest follow-up. On the other hand, of the patients with deterioration, 16 patients had a decreased intermittent claudication distance more than 50 m and 10 patients had newly developed or progressive paralysis. Subsequently, 19 patients received surgical intervention. The patients who got worse of their symptoms after discharge and then were performed conservative treatment were 3 patients of 26 patients with deterioration. The inpatient conservative treatment also consisted of pelvic traction, the application of a body cast, and an epidural steroid injection such as an epidural or selective nerve root block as described before. All these 3 patients received conservative treatment only once; however, 2 patients of them received surgical intervention after the second conservative treatment. Although the probability of symptom preservation after the successful conservative treatment of LSS gradually decreased over time, it remained 68.3% at 5 years post-discharge and 57.2% at 7 years post-discharge (Fig. 1). The number of patients who were able to follow up was 39 to 7 years after discharge. The outcomes of conservative treatment were worse in patients with severe intermittent claudication at the initial visit (≤ 100 m), cauda equina or mixed-type LSS, lower limb muscle weakness, and block on myelography than those in patients with mild intermittent claudication (between 100 and 500 m) (*p* = 0.004), radicular-type LSS (*p* = 0.039), no muscle weakness (*p* = 0.049), and no block on myelography (*p* = 0.045), respectively (Fig. 2a–d). The probability of symptom preservation at 5 years and median preservation expectancy in patients with severe intermittent claudication (≤ 100 m) were 38.9% and 4.1 years, respectively, whereas those in patients with mild intermittent claudication (between 100 and 500 m) were 81.1% and > 10 years. Similarly, in patients with the cauda equina and mixed types, the 5-year preservation probability and preservation expectancy were 52.4% and 5.8 years, respectively, whereas those in patients with the radicular type were 76.9% and 9.5 years. On the other hand, the 5-year preservation probabilities in patients with and without muscle weakness were 63.6% and 71.1%, although preservation expectancies were 5.8 years and > 10 years. In patients with and without block on myelography, the 5-year preservation probabilities were 60.0% and 74.3%, whereas preservation expectancies were 5.8 years and 9.5 years (Table 2). Age, vertebral body slip (≥ 3 mm), scoliosis (Cobb angle ≥ 10°), and redundant nerve roots of the cauda equina did not significantly influence symptom deterioration (Fig. 3a–d). Logistic regression analysis demonstrated that only severe intermittent claudication at the initial visit (≤ 100 m) was a significant risk factor incurring a poor outcome even after successful conservative treatment for LSS (*p* = 0.005, odds ratio = 6.665, correlation coefficient = 0.492) (Table 3). A post hoc power analysis demonstrated that the present study was enough powered with a power of 0.861 to detect differences between the patients with poor prognosis and good prognosis and that required sample size was 16/38 patients for the patients with poor/good prognosis, respectively.

**Table 1:** Patient population

	Preservation group	Deterioration group	*p* value
No. of patients	34	26	
Age (years)	62.5 ± 8.8	67.8 ± 6.9	0.015*
Male/female	11/23	15/11	0.068
Clinical classification			0.283
Radicular type	25	14	
Cauda equina type	5	7	
Mixed type	4	5	
Intermittent claudication			0.005*
Severe (≤ 100 m)	5	13	
Mild (> 100 m)	29	13	
Muscle weakness			0.029*
Presence	8	14	
Absence	26	12	
Vertebral body slip (≥ 3 mm)			0.596
Presence	17	9	
Absence	18	15	
Scoliosis (Cobb angle ≥ 10°)			0.491
Presence	7	3	
Absence	27	23	
Block on myelography			0.095
Completely blocked	24	11	
Not completely blocked	8	11	
Root defect	2	4	
Redundant nerve roots			0.044*
Presence	3	8	
Absence	31	18	

*Statistically significant

**Table 2:** Five-year preservation probability and median preservation expectancy

Factor	5-year preservation probability (%)	Median preservation expectancy (years)
Severe intermittent claudication	38.9	4.1
Mild intermittent claudication	81.1	Over 10
Cauda equina and mixed type of LSS	52.4	5.8
Radicular type of LSS	76.9	9.5
Muscle weakness	63.6	5.8
No muscle weakness	71.1	Over 10
Block on myelography	60.0	5.8
No block on myelography	74.3	9.5

**Table 3:** Logistic regression analysis of prognostic factors

Factor	Regression coefficient	Standard error	Odds ratio	*p* value	95% confidence interval
≤ 65 years or > 65 years	0.4954	0.6932	1.641	0.475	0.422–6.385
Clinical classification	0.7957	0.7122	2.216	0.264	0.549–8.950
Muscle weakness in lower limb	− 0.0176	0.7451	0.983	0.981	0.228–4.233
Intermittent claudication at the initial visit (severe)	1.8969	0.6817	6.665	0.005*	1.752–25.357
Vertebral body slip (3 mm or more)	− 0.5183	0.7214	0.596	0.473	0.145–2.449
Scoliosis (Cobb angle 10° or greater)	0.4510	0.9897	1.570	0.649	0.226–10.923
Block on myelography	0.8047	0.8126	2.236	0.322	0.455–10.994
Redundant nerve roots of cauda equina	− 0.4547	0.9992	0.635	0.649	0.090–4.498

*Statistically significant

## Discussion

Several reports have compared conservative and surgical treatments for LSS [14, 17, 18]. Amundsen et al. reported that the long-term results were better for surgery in a prospective 10-year follow-up study. However, more than half of the conservatively treated patients had a satisfactory outcome, and delaying surgery for some months did not worsen the prognosis. Therefore, a primarily conservative approach is recommended [14]. In their prospective 8- to 10-year follow-up study, Atlas et al. reported that low back pain relief, predominant symptom (either back or leg pain) improvement, and satisfaction were similar among patients who initially underwent surgery and those treated conservatively [17]. The randomized controlled trial study by Malmivaara et al. reported that patients treated both surgically and conservatively showed improved clinical outcomes in a 2-year follow-up [18]. Therefore, an initial conservative treatment approach seems advisable for appropriately selected patients.

Actually, we showed 56.7% of LSS patients treated by inpatient intensive conservative treatment maintained their condition at discharge for at least 5 years, average 7.3 years follow-up. Although there have been differences in evaluation methods or primal symptom severity, the reported outcomes of conservative treatment for LSS are generally good [7–10]. Johnson et al. reported 32 LSS patients who had conservative treatment, and the mean follow-up period was 4.1 years. Fifteen percent improved, 70% remained unchanged, and 15% deteriorated as assessed by the evaluation with the visual analog scale while 47% improved, 37% remained unchanged, and 16% deteriorated as assessed by the evaluation with their clinical examination [7]. Swezey reported 36 LSS patients who had conservative treatment by intermittent traction and/or epidural steroid injection, and the mean follow-up period was 5 years. Forty-three percent improved and 30% remained unchanged as assessed by a subjective assessment by using a telephone interview [10]. Miyamoto et al. reported 120 LSS patients who had conservative treatment by pelvic traction, application of body cast, and/or epidural steroid injection, and the mean follow-up period was 7.9 years. Forty-three percent improved, 17% remained unchanged, and 40% deteriorated as assessed by using a general evaluation with a modified JOA score [8]. Simotas et al. reported 49 LSS patients who had conservative treatment by exercise and analgesics and/or epidural steroid injection, and the mean follow-up period was 2.8 years. Forty-seven percent improved, 24% remained unchanged, and 29% deteriorated as assessed by using the pain/function score [9]. Our inpatient intensive conservative treatment was applied to the patients who failed to get satisfied with symptom relief by outpatient conservative treatment and demonstrated to be effective even for such persistent cases. Therefore, we believe that such inpatient conservative treatment should be positioned between outpatient conservative treatment and surgery.

The predictive factors for the prognosis of conservative treatment for LSS have been investigated in several studies [8, 9, 14, 15]. Although Herno et al. [15] and Amundsen et al. [14] failed to identify any predictive factors, Simotas et al. referred to the presence of degenerative scoliosis and advanced age as negative factors for the outcomes of conservative treatment [9], and Miyamoto et al. cited degenerative scoliosis as a negative factor in outcomes and reported that radicular LSS was a positive factor for outcomes [8]. However, these reports described only the prognostic factors at final follow-up and lacked the information regarding the time course and risk factors of symptom deterioration. It is indispensable to know when and how many patients experienced symptom deterioration after successful conservative treatment for making a decision whether or not to have surgery without trying intensive conservative treatment.

Our current study revealed prognostic factors and detailed clinical course of the symptom deterioration in LSS patients who once experienced symptom relief. Severe intermittent claudication at the initial visit (≤ 100 m), cauda equina or mixed type, lower limb muscle weakness, and block on myelography were identified as risk factors for symptom deterioration. Among them, severe intermittent claudication (≤ 100 m) was the only risk factor for symptom deterioration within 5 years post-discharge. In patients with severe intermittent claudication at the initial visit (≤ 100 m), deterioration was seen in 0.0% of patients by 3.6 years and increased to 61.2% by 4.3 years after discharge. Hence, surgery may be considered for patients with severe intermittent claudication (≤ 100 m) since its symptoms are indicative of deterioration from an early stage after discharge even if they temporarily improved. Conversely, patients with mild claudication (between 100 and 500 m) should not require early surgical treatment since symptom preservation would be expected for 10 years post-discharge. Although the long-term outcomes of cauda equina and mixed LSS were worse than those of radicular LSS, cauda equina and mixed LSS were not identified as risk factors of a poor outcome. Actually, patients in cauda equina and mixed LSS were more likely to feature severe intermittent claudication (9 out of 21 patients, 42.9%) than those in radicular LSS (9 out of 39 patients, 23.1%) even though there was no statistical significance (*p* = 0.110). Therefore, it is considered that there was a potential confounder between the clinical classification and severity of LSS, and classification of LSS did not affect the long-term outcome after conservative treatment of LSS directly but had indirect influence through the severity of intermittent claudication. Interestingly, in patients with lower limb muscle weakness and block on myelography, there were relatively small differences in preservation probability from those of patients without these features until 5 years post-discharge despite significant differences in long-term outcomes. The differences in long-term outcomes between patients with and without muscle weakness or block on myelography should depend on symptom deterioration after 5 years of follow-up. Regular follow-up for > 5 years after discharge in these patients is important; otherwise, surgery can be considered for patients with a life expectancy > 5 years.

### Limitations

The present study has some limitations. The largest limitation to this study is the relatively small sample size. We had only 60 patients followed longer than 5 years after successive conservative treatment of LSS. Although the power analysis showed strong statistical power, the sample size might be still insufficient to make conclusive statements. Therefore, our conclusion should be considered provisional and needs to be supported by a study with a larger patient population. Second, a mixed degree of symptom was included. The symptom at discharge after successful conservative treatment varied among patients because it depends a lot on the patients’ satisfaction, activity of daily life, or living environment. However, subjective evaluation by patients should be one of the most important outcomes. In the future study, it would be advisable to perform a survey using patient self-reporting measurement. Third, patients who had symptoms of LSS due to lumbar disc herniation may have been included, so the findings of these patients may be different. Lastly, more detailed radiological analysis such as lumbar canal diameters and quantification of the stenosis was not investigated. So, in the future, these factors should be investigated as prognostic factors of LSS after successful conservative treatment.

## Conclusions

Here, we investigated the prognosis of patients with LSS after successful conservative treatment. Cauda equina or mixed type, severe intermittent claudication at the initial visit (≤ 100 m), lower limb muscle weakness, and the block on myelography were the prognostic factors predicting the risk of aggravation. Surgery should be considered for patients with severe intermittent claudication.

## Abbreviation

LSS: Lumbar spinal canal stenosis
